# The associations of PON1 and APOE polymorphisms with plasma lipid levels and the risk for late complications in type 2 diabetes patients

**DOI:** 10.5937/jomb0-43154

**Published:** 2023-10-27

**Authors:** Jasna Klen, Katja Goričar, Vita Dolžan

**Affiliations:** 1 University Medical Centre Ljubljana, Department of Abdominal Surgery, Division of Surgery, Ljubljana, Slovenia; 2 University of Ljubljana, Faculty of Medicine, Ljubljana, Slovenia; 3 University of Ljubljana, Faculty of Medicine, Institute of Biochemistry and Molecular Genetics, Pharmacogenetics Laboratory, Ljubljana, Slovenia

**Keywords:** type 2 diabetes, hyperlipidemia, statins treatment, PON1, APOE, genetic polymorphisms, dijabetes tipa 2, hiperlipidemija, lečenje statinima, PON1, APOE, genetski polimorfizmi

## Abstract

**Background:**

Besides good glycemic control, also control of lipid levels can effectively prevent or delay late type 2 diabetes (T2D) complications. As apolipoprotein E (APOE) and paraoxonase 1 (PON1) were shown to suppress atherosclerosis, we investigated the associations of common functional PON1 and APOE polymorphisms with plasma lipid levels and the risk for late complications in T2D patients.

**Methods:**

Our retrospective genetic association study included 181 T2D patients genotyped for PON1 rs622, PON1 rs854560, APOE rs429358 and APOE rs7412.

## Abstract (sr)

**Metode:**

Na{a retrospektivna studija genetičke asocijacije uključila je 181 pacijenta sa T2D genotipiziranim za PON1 rs622, PON1 rs854560, APOE rs429358 i APOE rs7412.

**Rezultati:**

Nosioci alela PON1 rs622 G imali su značajno niži LDL-C (p=0,024) i niži odnos LDL/HDL (p=0,031) pod recesivnim modelom. Takođe u podgrupi pacijenata lečenih statinima, nosioci alela PON1 rs622 G imali su značajno niži LDL-C (p=0,019), niži odnos ukupnog holesterola/LDL (0,043) i niži odnos LDL/HDL (0,009) u poređenju sa onima koji nisu nosioci. Nakon prilagođavanja kliničkih karakteristika, ostala je značajna samo povezanost PON1 rs622 sa makrovaskularnim komplikacijama (p=0,002) i IM (p= 0,041). Genotipovi PON1 rs854560 i APOE nisu bili povezani sa kasnim komplikacijama T2D.

**Zaključak:**

Iako nasi podaci pokazuju neke povezanosti izmđu polimorfizma PON1 i nivoa lipida kod pacijenata sa T2D, kao i sa kasnim komplikacijama T2D, čini se da ove veze nisu klinički relevantne kod pacijenata sa T2D.

## Introduction

Type 2 diabetes (T2D) is a heterogeneous metabolic diseases in which complex genetic and environmental factors contribute to a progressive loss of mass and/or function of β-cell. Patients with T2D have insulin deficiency and peripheral insulin resistance which are characterised by elevated levels of blood glucose [1]. The incidence of T2D is rising dramatically and along it also the incidence of late complications, both microvascular such as retinopathy, nephropathy and peripheral neuropathy, as well as macrovascular complications such as peripheral arterial occlusive disease (PAOD), ischemic cerebrovas cular disease (ICD), and myocardial infarction (MI) [2] [3]. Macrovascular complications are the leading cause of morbidity and mortality in T2D patients. Good glycemic control delays microvascular complications, but prevention of macrovascular complications is still debatable. The incidence of cardiovascular diseases (CVD) is two to four times higher and they occur at a younger age in T2D patients compared to non-diabetic population. According to the latest data, the prevalence of CVD and atherosclerotic diseases is 34.8% (95% CI 32.7–36.8) and 31.8% (95% CI 29.7–33), respectively [4]. It was shown that besides good glycemic control, also control of lipid levels can effectively prevent late T2D complications [5]. It is generally accepted that statins which reduce cholesterol levels with inhibition of the HMG-CoA reductase enzyme should be used in primary and secondary prevention of CVD [5].

Cholesterol is transported by lipoproteins, the most cholesterol rich lipoproteins being low density lipoproteins (LDL) and high density lipoproteins (HDL). It is generally accepted that the imbalance between LDL and HDL contained cholesterol (LDL-C and HDL-C, respectively) is associated with increased risk of cardiovascular events such as MI and ICD [6].

One of the factors involved in lipoprotein transport and metabolism is glycoprotein called apolipoprotein E (APOE), which is a high-affinity ligand for very low density lipoproteins (VLDL) receptors, LDL receptors related protein and LDL receptors [6] [7]. APOE is synthesized by the liver and can be found in three isoforms (APOE2, APOE3, APOE4) with differing amino acid residues, all of which exhibit specific effects [8]. APOE was reported to suppress atherosclerosis and has also other protective functions such as inhibition of platelet aggregation, anti-inflammatory effects and regulation of microRNA levels [9]. Recent studies found that in hyperlipidemic mice apoE suppress myelopoeisis [10] and proliferation and activation of monocytes [11].

APOE gene is located on chromosome 19q13.2. Two APOE polymorphisms, rs429358 (p.Cys112Arg) and rs7412 (p.Arg158Cys) are common in population and define three polymorphic alleles APOE2, APOE3 and APOE4 that encode three respective protein variants: APOE2 (Cys112, Cys158), APOE3 (Cys112, Arg158), and APOE4 (Arg112, Arg158) [12]. Among all populations, APOE3 allele is the most frequent (50–90%), followed by APOE4 (5–35%) and APOE2 allele (1–5%). Substitution of one or two amino acids affects the total charge and structure of APOE, leading to alteration in binding to cellular receptors and lipoprotein particles and possibly changing the stability and rate of production and clearance [13].

The role of APOE in catabolism of lipoproteins is well-studied [14]. APOE regulates their metabolism through binding to APOE receptors, directing the transport, delivery, and distribution of lipoproteins [13]. The common APOE variations account for around 4% of the variability in plasma cholesterol levels. Several studies found higher incidence of T2D and CVD in APOE4 carriers and suggested that this may be associated with higher total plasma cholesterol levels [15] [16] [17].

Another significant factor that may influence lipid metabolism in T2D is the paraoxonase (PON) family, including PON1, PON2 and PON3. PON1 is produced mainly in the liver, then secreted into the blood, where it physically binds to the HDL particles. PON1 is a multifunctional enzyme with several enzymatic activities. It also has the ability of reducing the oxidated low-density lipoproteins (Ox-LDL) and modulating cholesterol efflux from macrophages and reducing endo thelial injury and vascular inflammation [18]. Although ApoA-I is known as the main antioxidant component in HDL, PON1 may also contribute to antioxidant and antiatherogenic properties of HDL [19].

Five functional polymorphisms are recognized to influence PON1 concentration and activity. Two are located in the encoding region of the gene. The PON1 rs662 polymorphism results in substitution of glutamine with arginine (p.Gln192Arg), where the Gln192 isoform expresses greater protective properties against oxidized LDL metabolism than the Arg192 isoform. The other coding region polymorphism is rs854560 that leads to the substitution of leucine by methionine (p.Leu55Met) [20]. Polymorphic allele was associated with lower PON1 activity in comparison to the wild-type genotype [21].

We investigated the associations of common functional PON1 and APOE polymorphisms with plasma lipid levels and the risk for late complications in T2D patients.

## Materials and methods

Our retrospective genetic association study included T2D patients who came for their regular follow up visits at the outpatient clinic at the General Hospital Trbovlje. The majority of patients was treated with metformin either in monotherapy or in combination with sulphonylureas, with the exception of 25 patients with end-stage renal failure due to diabetic nephropathy that were treated with insulin. A total of 124 T2D patients had hyperlipidemia and were also treated with statins.

Patients with heart failure, active cancer, type 1 diabetes (T1DM), gestational diabetes, other types of diabetes, conditions that can cause hyperglycaemia, co-treatment with corticosteroids, dementia or severe psychiatric disorders, addiction to alcohol or illegal drugs were excluded from the sample as described in detail in our previous studies [22] [23].

Data on the history of diabetes, presence of arterial hypertension, hyperlipidemia and late complications, smoking status and other medications were collected from the interview at the inclusion in the study and also from their medical records. A complete physical examination and laboratory evaluation was performed upon the entering of the study. An evaluation of blood pressure, body weight and height was performed at each follow-up and body mass index (BMI) was calculated accordingly. Plasma lipid levels as well as kidney function (urea and creatinine, urine albumin and albumin/creatinine ratio) were assessed on a yearly basis. All the laboratory parameters were measured using standard laboratory procedures at the biochemistry laboratory of the General Hospital Trbovlje, Slovenia. Once a year screening for diabetic retinopathy was performed by an ophthalmologist. Echosonography and exercise stress test (cycloergometry) were conducted both at the first visit and in case of any symptoms suggestive for ischemic heart disease [23].

The study was approved by the National Ethics Committee and conducted in accordance with the Declaration of Helsinki. Written informed consent was obtained from all subjects.

DNA extraction and genotyping analyses were performed at the Pharmacogenetics Laboratory, Institute of Biochemistry, Faculty of Medicine, University of Ljubljana. Genomic DNA was isolated from peripheral blood leukocytes using Qiagen Flexi-Gene kit (Qiagen, Hilden, Germany). Genotyping of PON1 rs622, PON1 rs854560, APOE rs429358 and APOE rs7412 was carried out using a fluorescence-based competitive allele-specific polymerase chain reaction (KASPar) assay according to the manufacturer's instructions (KBiosciences, Herts, UK).

### Statistical analysis

Continuous and categorical variables were described using median with 25%–75% range and frequencies, respectively. Both additive and dominant genetic model were used in the analyses. Deviation from Hardy-Weinberg equilibrium (HWE) was evaluated using chi-square test. Nonparametric Mann-Whitney test or Kruskal-Wallis test with post hoc Bonferroni corrections for pairwise comparisons were used to compare the distribution of continuous variables between two or more groups, respectively. To evaluate the association of selected polymorphisms with late complications, univariable and multivariable logistic regression was used to calculate the odds ratios (ORs) and 95% confidence intervals (CIs). Clinical parameters used for adjustment in multivariable analysis were selected using stepwise forward-conditional logistic regression. Fisher's exact test was used if there were no patients in one of the categories and for comparison of categorical variables between groups. All statistical tests were two-sided and the level of significance was set at 0.05. The statistical analyses were carried out by using IBM SPSS Statistics version 27.0 (IBM Corporation, Armonk, NY, USA).

## Results

Our study included 181 T2D patients, 105 (58.0%) male and 76 (42.0%) female. With regards to requirement of hyperlipidemia treatment, 124 (68.5%) patients were treated with statins, while 57 (31.5%) did not require statin treatment to have well controlled plasma lipid levels. Macrovascular complications were diagnosed in 46 (25.4%) patients, 36 (29.0%) of which were treated with statins and 10 (17.5%) were not. A total of 32 (17.7%) patients suffered MI, 27 (21.8%) of them receiving statins and 5 (8.8%) were not. Only 10(5.5%) patients of the total sample had PAOD, out of which 8 (6.5%) were on statin treatment and 2 (3.5%) were not. In addition 16 (8.85%) patients suffered from ICD, among them 12 (9.7%) were receiving statin treatment and 4 (7.0%) were not. Microvascular complications were diagnosed in 34 (18.8%) patients, 8 (14.0%) of which were treated with statins and 26 (21.0 %) were not receiving statins. A total of 25 (13.8%) patients had end-stage kidney failure, 15 (8.3%) retinopathy and 13 (7.2%) neuropathy. Patients’ characteristicsare shown in Table 1. Genotype frequencies of the investigated polymorphisms are shown in Supplementary Table 1. All genotype frequencies were consistent with HWE (p>0.05). No significant difference was observed in the frequency distribution of the investigated genotypes among T2D patients with or without statin treatment (Supplementary Table 1).

**Table 1:** Clinical characteristics of T2D patients Data are presented as median (25%–75%) for continuous variables and as N (%) for categorical variables.<br>*data available only for 104 T2D patients<br>#Comparison between patients not treated with statins and patients with statins using Mann Whitney test for continuous variables and Fisher’s exact test for categorical variables<br>BMI, body mass index; HDL, high density lipoproteins; ICD, ischemic cerebrovascular disease; LDL, low density lipoproteins; MI, myocardial infarction; PAOD, peripheral arterial occlusive disease; T2D, type 2 diabetes, TAG, triacylglycerols

Characteristic	All patient<br> s (N=181)	Patients not treated<br> with statins<br> (N=57)	Patients treated<br> with statins<br> (N=124)	p#
Sex				
Male	105 (58.0)	30 (52.6)	75 (60.5)	0.335
Female	76 (42.0)	27 (47.4)	49 (39.5)	
Age (years)	64.0 (58.5–70.5)	64 (56.5–70)	64.5 (60–71)	0.291
Duration of T2D (years)	11.0 (6.0–17.0)	11 (5–16)	12 (7–20)	0.233
HbA1c (%)	6.9 (6.3–7.6)	6.7 (6.3–7.7)	6.9 (6.4–7.6)	0.678
Basal glucose (mmol/L)*	7.5 (6.7–8.7)	7.7 (6.6–8.9)	7.4 (6.7–8.6)	0.511
BMI (kg/m^2^)	30.0 (28.0–33.3)	29 (28–32.5)	30 (28–34)	0.436
Systolic blood pressure (mmHg)	135 (130–145)	135 (130–140)	138 (130–149)	0.383
Diastolic blood pressure (mmHg)	80 (70–80)	80 (72–80)	80 (70–80)	0.260
Total cholesterol (mmol/L)	4.2 (3.5–5.0)	4.7 (3.6–5.6)	4 (3.5–4.9)	0.009
LDL cholesterol (mmol/L)	2.4 (1.9–3.1)	2.7 (2–3.6)	2.3 (1.8–2.8)	0.001
HDL cholesterol (mmol/L)	1.1 (1.0–1.4)	1.1 (1–1.4)	1.1 (1–1.4)	0.897
TAG (mmol/L)	1.6 (1.2–2.4)	1.5 (1.1–2.2)	1.7 (1.2–2.5)	0.181
Total cholesterol/HDL ratio	3.6 (2.8–4.3)	3.9 (3–4.9)	3.5 (2.7–4.2)	0.047
LDL/HDL ratio	2.1 (1.5–2.7)	2.3 (1.7–3.3)	1.9 (1.4–2.5)	0.003
Macrovascular complications	46 (25.4)	10 (17.5)	36 (29.0)	0.141
PAOD	10 (5.5)	2 (3.5)	8 (6.5)	0.509
ICD	16 (8.8)	4 (7.0)	12 (9.7)	0.779
MI	32 (17.7)	5 (8.8)	27 (21.8)	0.037
Microvascular complications	34 (18.8)	8 (14.0)	26 (21.0)	0.311
End-stage kidney failure	25 (13.8)	6 (10.5)	19 (15.3)	0.489
Retinopathy	15 (8.3)	2 (3.5)	13 (10.5)	0.151
Neuropathy	13 (7.2)	2 (3.5)	11 (8.9)	0.232

In the entire group of patients, carriers of two polymorphic PON1 rs622 G alleles had significantlylower LDL-C (p=0.024) and lower LDL/HDL ratio (p=0.031) (Table 2). These associations remainedsignificant in the subgroup of patients treated with statins. Statin treated T2D patients carrying two polymorphic PON1 rs622 G alleles had significantly lower LDL-C (p=0.019), lower total cholesterol/LDL ratio (0.043) and lower LDL/HDL ratio (0.009). However in patients not treated with statins, the only significant association of PON1 rs662 was observed with higher HDL-C levels under the dominant genetic model (p=0.023) (Table 3).

**Table 2:** Association of PON1 polymorphisms with T2D patients’ clinical characteristics (N=181) *pairwise comparisons: AG vs AA: P = 0.032; **pairwise comparisons: GG vs AG: P = 0.022; ***pairwise comparisons: GG vs AG: P = 0.025<br>BMI, body mass index; HDL, high density lipoproteins; LDL, low density lipoproteins; T2D, type 2 diabetes, TAG, triacylglycerols

Characteristic	PON1 rs854560						PON1 rs622					
	AA	AT	TT	P	AT+TT	P	AA	AG	GG	P	AG+GG	P
HbA1c (%)	6.9<br>(6.3–7.6)	6.9<br> (6.3–7.6)	6.7 (6.4–7.2)	0.885	6.8 (6.4–7.6)	0.720	6.8 (6.4–7.6)	6.7 (6.2–7.4)	7.3 (6.7–8)	0.076	6.8 (6.3–7.6)	0.814
Basal glucose<br> (mmol/L)	7.2 (6.7–8)	7.7 (7–9)	7.2 (6.6–8.9)	0.085	7.7 (6.7–8.9)	0.058	7.4 (6.6– 8.7)	7.6 (6.9–8.7)	7.1 (6.2–8.8)	0.675	7.5 (6.9–8.8)	0.639
BMI (kg/m^2^)	30<br> (27.9–33.1)	29<br> (27.8–34.5)	29<br> (28–33)	0.821	29<br> (28–34)	0.709	29<br> (28–32)	31<br> (28–35.3)	30<br> (27.5–36)	0.037*	31<br> (28–35.5)	0.012
Systolic blood<br> pressure (mmHg)	135<br> (130–145)	140<br> (130–145)	135<br> (119–143)	0.675	138<br> (125–145)	0.703	140<br> (125–150)	135<br> (130–144)	135<br> (130–160)	0.881	135<br> (130–145)	0.885
Diastolic blood<br> pressure (mmHg)	80<br> (70–80)	80<br> (70–80)	80<br> (74–80)	0.990	80<br> (70–80)	0.926	80<br> (70–80)	80<br> (72–80)	75<br> (70–85)	0.701	80<br> (70–80)	0.988
Total<br> cholesterol<br> (mmol/L)	4.2<br> (3.4–5.2)	4.2<br> (3.7–5.2)	4.1<br> (3.4–4.8)	0.520	4.1<br> (3.6–5)	0.750	4.1<br> (3.6–5)	4.5<br> (3.7–5.3)	3.5<br> (3.1–4.3)	0.088	4.3<br> (3.5–5.3)	0.582
LDL cholesterol<br> (mmol/L)	2.4<br> (1.7–3.1)	2.4<br> (1.9–3.2)	2.2<br> (1.9–2.7)	0.622	2.4<br> (1.9–3)	0.539	2.3<br> (1.9–3)	2.5<br> (1.9–3.3)	1.8<br> (1.2–2.5)	0.024**	2.4<br> (1.8–3.2)	0.637
HDL cholesterol<br> (mmol/L)	1.2<br> (1–1.4)	1.1<br> (1–1.5)	1<br> (1–1.3)	0.239	1.1<br> (1–1.4)	0.342	1.1<br> (1–1.3)	1.2<br> (1–1.4)	1.3<br> (1.1–1.5)	0.300	1.2<br> (1–1.4)	0.359
TAG (mmol/L)	1.7<br> (1.3–2.4)	1.5<br> (1.1–2.4)	1.7<br> (1.1–2.3)	0.464	1.5<br> (1.1–2.3)	0.229	1.5<br> (1.1–2.4)	1.6<br> (1.3–2.2)	1.7<br> (1.1–2.5)	0.897	1.6<br> (1.3–2.4)	0.699
Total cholesterol/<br>HDL ratio	3.5<br> (2.8–4.3)	3.8<br> (2.8–4.3)	3.6<br> (2.7–4.8)	0.692	3.7<br> (2.7–4.4)	0.400	3.7<br> (2.7–4.5)	3.6<br> (3.2–4.3)	2.8<br> (2.2–4.2)	0.085	3.6<br> (2.8–4.3)	0.918
LDL/HDL ratio	1.9<br> (1.4–2.6)	2.2<br> (1.5–2.7)	2.1<br> (1.4–2.9)	0.521	2.2<br> (1.5–2.7)	0.254	2.1<br> (1.4–2.7)	2.2<br> (1.8–2.7)	1.3<br> (0.8–2.1)	0.031***	2.1<br> (1.5–2.7)	0.913

**Table 3:** Association of PON1 rs622 polymorphism with patients clinical characteristics in T2D patients not treated with statins and patients treated with statins *pairwise comparisons: GG vs AA: P = 0.046, GG vs AG: P = 0.015; **pairwise comparisons: GG vs AG: P = 0.037; ***pairwise comparisons: GG vs AA: P = 0.025, GG vs AG: P = 0.006.<br>BMI, body mass index; HDL, high density lipoproteins; LDL, low density lipoproteins; T2D, type 2 diabetes, TAG, triacylglycerols

Characteristic	Patients not treated with statins(N=57)						Patients treated with statins(N=124)					
	PON1 rs622						PON1 rs622					
	AA	AG	GG	P	AG+GG	P	AA	AG	GG	P	AG+GG	P
HbA1c (%)	6.8<br> (6.4–7.5)	6.6<br> (6.2–7.9)	7.1<br> (6.7–7.8)	0.592	6.6<br> (6.2–7.9)	0.942	6.8<br> (6.4–7.6)	6.8<br> (6–7.4)	7.5<br> (6.7–8.1)	0.120	6.9<br> (6.3–7.5)	0.884
Basal glucose<br> (mmol/L)	8.2<br> (6.6–9)	7.4<br> (6.9–8.9)	7.1<br> (7.1–)	0.780	7.2<br> (6.9–8.8)	0.599	7.3<br> (6.6–8.6)	7.7<br> (6.9–8.2)	7.7<br> (5.4–8.9)	0.520	7.7<br> (6.9–8.8)	0.309
BMI (kg/m^2^)	29<br> (28–32)	30<br> (28–37)	27.5<br> (22.6–29)	0.150	29<br> (27–35)	0.488	29<br> (27.4–32.1)	32<br> (28–35)	30.5<br> (29–36)	0.037	31.8<br> (28.8–36)	0.011
Systolic blood<br> pressure<br> (mmHg)	135<br> (124–140)	135<br> (130–145)	133<br> (130–154)	0.681	135<br> (130–145)	0.382	140<br> (130–150)	135<br> (130–143)	135<br> (125–160)	0.828	135<br> (130–145)	0.766
Diastolic<br> blood pressure<br> (mmHg)	80<br> (70–80)	80<br> (75–85)	73<br> (63–79)	0.151	80<br> (73–82)	0.656	80<br> (70–80)	80<br> (70–80)	75<br>(70–90)	0.914	80<br> (70–80)	0.724
Total cholesterol<br> (mmol/L)	4.5<br> (3.5–5.6)	4.9<br> (4–5.5)	4.5<br> (3.2–6.3)	0.547	4.9<br> (3.8–5.6)	0.283	4.1<br> (3.7–4.9)	4<br> (3.5–5.2)	3.5<br> (3.1–4.3)	0.135	3.9<br> (3.4–4.9)	0.685
LDL cholesterol<br> (mmol/L)	2.7<br> (1.9–3.7)	2.9<br> (2.2–3.5)	3<br> (1.5–4.5)	0.940	2.9<br> (2.2–3.5)	0.724	2.3<br> (1.9–2.7)	2.4<br> (1.8–3)	1.7<br> (1.1–2.1)	0.019*	2.3<br> (1.7–2.9)	0.890
HDL cholesterol<br> (mmol/L)	1<br> (1–1.3)	1.3<br> (1–1.6)	1.3<br> (1.2–1.4)	0.073	1.3<br> (1–1.5)	0.023	1.1<br> (1–1.4)	1.1<br> (0.9–1.4)	1.4<br> (1–1.7)	0.362	1.2<br> (0.9–1.4)	0.731
TAG (mmol/L)	1.9<br> (1–2.4)	1.4<br> (1–1.7)	1.5<br> (1.1–2.1)	0.736	1.4<br> (1.1–1.7)	0.437	1.5<br> (1.1–2.4)	1.8<br> (1.3–2.7)	2.1<br> (1.1–2.5)	0.526	1.8<br> (1.3–2.6)	0.261
Total<br> cholesterol/<br>HDL ratio	4.3<br> (2.8–5.3)	3.7<br> (3.2–4.6)	3.6<br> (2.5–4.8)	0.672	3.7<br> (3.2–4.6)	0.405	3.5<br> (2.6–4.2)	3.6<br> (2.9–4.3)	2.5<br> (2.1–3.5)	0.043**	3.5<br> (2.8–4.2)	0.849
LDL/HDL ratio	2.7<br> (1.5–3.6)	2.2<br> (1.8–3)	2.2<br> (1.3–3.4)	0.633	2.2<br> (1.8–3.1)	0.349	1.9<br> (1.4–2.5)	2<br> (1.6–2.6)	1.1<br> (0.8–1.7)	0.009***		0.933

APOE genotypes were not associated with any of the patient's characteristics in the whole study group, except for the basal glucose levels, which were higher in carriers of at least one rs7412 polymorphic allele (8.7 (7.1–9.8) vs 7.3 (6.7–8.2) mmol/L, p = 0.017). When the patients were stratified according to statin treatment, rs7412 was only associated with higher basal glucose in patients treated with statins (p = 0.014, Supplementary Table 2). On the other hand, rs429358 was associated with higher basal glucose only in patients not treated with statins (p = 0.006, Supplementary Table 2). The only association between APOE and plasma lipids was observed for carriers of at least one rs7412 polymorphic allele and higher TAG levels in patients treated with statins (p=0.035) (Supplementary Table 2).

Regarding late complications, PON1 rs622 GG genotype was associated with increased risk for macro vascular complications (OR=5.4, 95% CI=1.72–16.98, p=0.004), in particular with MI (OR=4.49, 95%CI=1.41–14.26, p=0.011). Among microvascular complications, rs622 GG genotype was associated with increased risk for retinopathy (OR=2.48, 95% CI=p=0.022), while carriers of at least one G allele had an increased risk of end-stage renal disease (OR=5.21, 95% CI=1.27–21.4, p=0.047). After adjustment for clinical characteristics only associations of PON1 rs622 with macrovascular complications (OR=8.61, 95%CI=2.23–33.27, p=0.002) and MI (OR=3.74, 95%CI=1.06–13.21, p=0.041) remained significant (Table 4).

**Table 4:** Association of PON1 rs622 with late complications in T2D patients *calculated using Fisher’s exact test<br>**adjustment for clinical variables, macrovascular complications: T2D duration and HDL; PAOD: age, HbA1c, and TAG; ICD: total cholesterol; MI, microvascular complications, neuropathy: T2D duration; end-stage kidney failure: T2D duration, diastolic blood pressure,total cholesterol and TAG; retinopathy: T2D duration and LDL<br>HDL, high density lipoproteins; ICD, ischemic cerebrovascular disease; LDL, low density lipoproteins; MI, myocardial infarction; PAOD, peripheral arterial occlusive disease; T2D, type 2 diabetes, TAG, triacylglycerols

		*PON1* rs622				*PON1* rs622 – adjusted for clinical risk factors**			
		AA	AG	GG	AG+GG	AA	AG	GG	AG+GG
Macrovascular<br>complications	N (%)	20 (21.7)	17 (23.0)	9 (60.0)	26 (29.2)				
	OR<br>(95 % CI)	Ref.	1.07<br>(0.52–2.24)	5.4<br>(1.72–16.98)	1.49<br>(0.76–2.91)	Ref.	0.98<br>(0.45–2.15)	8.61<br>(2.23–33.27)	1.43<br>(0.69–2.94)
	P		0.849	0.004	0.250		0.995	0.002	0.335
PAOD	N (%)	6 (6.5)	4 (5.4)	0 (0.0)	4 (4.5)				
	OR<br>(95 % CI)	Ref.	0.82<br>(0.22–3.02)	/	0.67<br>(0.18–2.48)	Ref.	0.48<br>(0.11–2.11)	/	0.42<br>(0.10–1.81)
	P		0.764	0.562*	0.553		0.330		0.243
ICD	N (%)	8 (8.7)	5 (6.8)	3 (20.0)	8 (9.0)				
	OR<br>(95 % CI)	Ref.	0.76<br>(0.24–2.43)	2.62<br>(0.61–11.28)	1.04<br>(0.37–2.89)	Ref.	0.83<br>(0.25–2.69)	2.18<br>(0.49–9.67)	1.08<br>(0.38–3.06)
	P		0.645	0.195	0.945		0.752	0.306	0.891
MI	N (%)	15 (16.3)	10 (13.5)	7 (46.7)	17 (19.1)				
	OR<br>(95 % CI)	Ref.	0.8<br>(0.34–1.91)	4.49<br>(1.41–14.26)	1.21<br>(0.56–2.6)	Ref.	0.68<br<(0.27–1.69)	3.74<br>(1.06–13.21)	1.01<br>(0.45–2.27)
	P		0.618	0.011	0.622		0.401	0.041	0.990
Microvascular<br>complications	N (%)	14 (15.2)	16 (21.6)	4 (26.7)	20 (22.5)				
	OR<br>(95 % CI)	Ref.	1.54<br>(0.69–3.4)	2.03<br>(0.56–7.27)	1.61<br>(0.76–3.44)	Ref.	1.38<br>(0.59–3.25)	1.31<br>(0.31–5.56)	1.37<br>(0.60–3.10)
	P		0.289	0.279	0.214		0.460	0.718	0.454
End-stage<br>kidney<br>failure	N (%)	8 (8.7)	13 (17.6)	4 (26.7)	17 (19.1)				
	OR<br>(95 % CI)	Ref.	2.24<br>(0.87–5.73)	3.82<br>(0.99–14.8)	2.48<br>(1.01–6.08)	Ref.	2.39<br>(0.78–7.29)	2.07<br>(0.38–11.23)	2.32<br>(0.80–6.68)
	P		0.093	0.053	0.047		0.127	0.397	0.120
Retinopathy	N (%)	6 (6.5)	5 (6.8)	4 (26.7)	9 (10.1)				
	OR<br>(95 % CI)	Ref.	1.04<br>(0.3–3.55)	5.21<br>(1.27–21.4)	1.61<br>(0.55–4.73)	Ref.	0.85<br>(0.20–3.70)	0.29<br>(0.44–19.30)	1.20<br>(0.32–4.48)
	P		0.952	0.022	0.385		0.831	0.265	0.781
Neuropathy	N (%)	7 (7.6)	4 (5.5)	2 (13.3)	6 (6.8)				
	OR<br>(95 % CI)	Ref.	0.7<br>(0.2–2.5)	1.87<br<(0.35–9.99)	0.89<br>(0.29–2.76)	Ref.	0.63<br>(0.17–2.29)	1.33<br>(0.23–7.78)	0.75<br>(0.23–2.41)
	P		0.588	0.465	0.838		0.479	0.750	0.631

PON1 rs854560 and APOE genotypes were not associated with late T2D complications (Supplementary Table 3 and Supplementary Table 4).

## Discussion

Our study assessed the associations of PON1 and APOE polymorphisms with plasma lipid levels and the risk for late complications in T2D patients either treated with statins or not.

Regarding the lipid levels, when all T2D patients were considered, carriers of at least one polymorphic PON1 rs622 G allele had significantly lower LDL-C, and lower LDL/HDL ratio. When T2D patients were divided in two groups based on statin treatment, these associations remained significant only in statin treated patients. In patients not treated with statins, PON1 rs622 polymorphism was associated with HDL-C levels, however the differences in plasma levels among different genotype groups were not clinically relevant. The association between PON1 rs622 G and total cholesterol/HDL-C ratio was significant only in statin-treated patients. In agreement with our data, the largest meta-analysis to date showed that PON1 rs662 G allele carriers had higher TAG, total cholesterol and LDL-C levels, but there was no association with plasma levels of HDL-C. However no data is available if the patients included in this meta-analysis were treated with statins. Published evidence suggests that there is no correlation between PON1 rs622 or PON1 rs854560 variant and lipoprotein concentration, although both investigated polymorphisms were shown to decrease the enzymatic activity of PON1 [18]. It was observed that PON1 activity correlated positively with total plasma APOE levels, however this association was present only in patients without metabolic syndrome (MetS), but not in patients with MetS or T2D [24]. This suggested that circulating APOE levels may positively influence PON1 activity, but that MetS related changes in the composition of HDL particles may decrease this effect [25]. However, a study including patients with and without T2D clearly showed that although PON1 activity was more related to HDL particle concentration than to HDL-C levels, T2D-associated impairment of PON1 activity was not attributable to alterations in HDL structure [26].

We observed no association between APOE polymorphisms and lipid levels, except for higher TAG levels in carriers of at least one polymorphic APOE rs7412 T allele. A study in Han Chinese patients reported significant associations between APOE2, APOE3, and APOE4 genotypes and the levels of LDL-C both in T2D patients and healthy individuals, however there was no significant association with TAG/HDL-C ratio [27].

We observed that among T2D patients carriers of PON1 rs622 GG genotype had higher incidence of macrovascular complications. Among them, the most prominent association was observed with MI, while there was no significant association with the risk for PAOD or ICD. A higher risk of MI was also reported in Han Chinese T2D or obese patients with the GA/AA genotype [28]. PON1 rs622 AA genotype was also associated with increased risk of coronary artery disease (CAD) as opposed to PON1 rs854560 TT genotype which was associated with decreased risk [29]. This association could be partly due to the oxidated-LDL levels and partly by abnormal lipid levels associated with the variant rs622 G allele [21]. However, Arca et al found no major effect of PON1 rs854560 polymorphism on CAD [30]. That relates well with our study findings which do not show any indication of an important association between PON rs854560 polymorphism and any late T2D complications.

Regarding the associations of PON1 with microvascular late complications, in our study sample, the presence of PON1 rs622 GG genotype also posed a higher risk of diabetic retinopathy. However another study observed an association between diabetic retinopathy and PON1 rs854560, but not PON1 rs622 [31]. We also observed a trend of association between PON1 rs622 AG/GG genotype and higher risk for diabetic nephropathy. In contrast with our data, *Murata et al*. [32] observed an association between diabetic nephropathy and PON1 rs622 AA genotype.

In our study, APOE genotypes were not associated with any of the late T2D complications. However, in Han Chinese population, APOE gene polymorphisms were associated with risk for CAD both in T2D patients and healthy individuals. In both groups, APOE allele E4 increased the risk of developing CAD, compared with the allele E3. This association remained significant also after adjustment for age and sex [27]. A meta-analysis of four studies also confirmed that APOE E4 polymorphism increases the risk of CAD in patients with T2D. This risk could perhaps be attributed to the significant differences in LDL-C plasma levels among T2D patients with different APOE isoforms. Furthermore, the HDL-C levels were lower in CAD patients with APOE E4 genotypes compared to homozygotes for E3/E3 genotype [27]. On the contrary, APOE E2/E3 genotype presented independent risk factor for developing ICD in T2D patients [33]. Another study confirmed the associations of APOE allele E4 with the risk for T2D, CHD in T2D, and ICD in T2D, and also indicated that APOE allele E2 may be a risk factor for diabetic nephropathy [34]. Although APOE polymorphism was not associated with T2D in central European Caucasian Czech population in a previous study, APOE4 allele was associated with the risk for diabetic retinopathy. In particular, female T2D patients with at least one APOE4 allele showed lower prevalence of retinopathy [35].

Our results need to be interpreted with caution due to the retrospective nature of our study and due to the relatively small patient sample with limited numbers of patients with late complications. Furthermore, our results could be affected by the fact that patients included in our study were treated with metformin and SU. Metformin may affect the levels of PON1 [21]. Indeed, increase in serum PON1 activity and reduction of oxidative stress markers were reported in patients with T2D [36]. Furthermore, in rat models, metformin [36] [37] increased PON1 expression levels in liver and serum levels, while treatment with sulfonylureas [38] increased PON1 activity in the liver, but not in plasma. As our study was designed as genetic association study, PON1 plasma levels and activity were not assessed, which in another limitation of our study.

## Conclusions

In conclusion, our data show some associations between PON1 polymorphisms and lipid levels in T2D patients, as well as with late T2D complications. On the other hand, despite some observed associations of APOE polymorphisms with plasma lipids, these associations do not seem to be clinically relevant in T2D patients.

## Dodatak

### Acknowledgments

We are grateful to Mrs. Savica Soldat from The Pharmacogenetics Laboratory for her expert technical assistance and Veronika Kra ek, MD for her assistance in drafting the first version of the paper.

### Funding

The study was supported by the Slovenian Research Agency (grants No P1-0170 and P3-0298).

### Conflict of interest statement

All the authors declare that they have no conflict of interest in this work.

### List of abbreviations

APOE, Apolipoprotein E;

Arg, arginine; BMI,body mass index;

CAD, coronary artery disease;

CI, confidence interval;

CVD, cardiovascular diseases;

Cys, cysteine;

Gln, glycine;

HDL, high density lipoproteins;

HDL-C, HDL contained cholesterol;

HMG-CoA, -Hydroxy-methylglutaryl-CoA;

HWE, Hardy-Weinberg equilibrium;

ICD, ischemic cerebrovascular disease;

LDL, low density lipoproteins;

LDL-C, LDL contained cholesterol;

Leu, leucine;

Met, methionine;

MetS, metabolic syndrome;

MI, myocardial infarction;

OR, odds ratio;

Ox-LDL, oxidated low-density lipoproteins;

PAOD, peripheral arterial occlusive disease;

PON1, Paraoxonase 1;

T1DM, type 1 diabetes;

T2D, type 2 diabetes;

TAG, triacylglycerols;

VLDL – very low density lipoproteins
